# Genome-wide association study identifies SNP markers and putative candidate genes for terpene traits important for *Leptocybe invasa* resistance in *Eucalyptus grandis*

**DOI:** 10.1093/g3journal/jkac004

**Published:** 2022-02-03

**Authors:** Lorraine Mhoswa, Alexander A Myburg, Bernard Slippers, Carsten Külheim, Sanushka Naidoo

**Affiliations:** 1 Department of Biochemistry, Genetics and Microbiology, Forestry and Agricultural Biotechnology Institute (FABI), University of Pretoria, Pretoria 0028, South Africa; 2 College of Forest Resources and Environmental Science, Michigan Technological University, Houghton, MI 49931-1295, USA

**Keywords:** *Eucalyptus grandis*, *Leptocybe invasa*, genome-wide association study, terpenes, single nucleotide polymorphism, multilocus mixed model, Genomic Prediction, GenPred, Shared Data Resource

## Abstract

Terpenes are an important group of plant specialized metabolites influencing, amongst other functions, defence mechanisms against pests. We used a genome-wide association study to identify single nucleotide polymorphism (SNP) markers and putative candidate genes for terpene traits. We tested 15,387 informative SNP markers derived from genotyping 416 *Eucalyptus grandis* individuals for association with 3 terpene traits, 1,8-cineole, γ-terpinene, and *p*-cymene. A multilocus mixed model analysis identified 21 SNP markers for 1,8-cineole on chromosomes 2, 4, 6, 7, 8, 9, 10, and 11, that individually explained 3.0%–8.4% and jointly 42.7% of the phenotypic variation. Association analysis of γ-terpinene found 32 significant SNP markers on chromosomes 1, 2, 4, 5, 6, 9, and 11, explaining 3.4–15.5% and jointly 54.5% of phenotypic variation. For *p*-cymene, 28 significant SNP markers were identified on chromosomes 1, 2, 3, 5, 6, 7, 10, and 11, explaining 3.4–16.1% of the phenotypic variation and jointly 46.9%. Our results show that variation underlying the 3 terpene traits is influenced by a few minor loci in combination with a few major effect loci, suggesting an oligogenic nature of the traits.

## Introduction

The foliage of many *Eucalyptus* trees is rich in a group of plant specialized metabolites known as terpenes (Boland *et al.* 1991). Terpenes are a highly diverse and dynamic class of chemicals with more than 20,000 known structures (Degenhardt *et al.* 2003). The major subclasses of terpenes includes, hemi- (C_5_), mono- (C_10_), sesqui- (C_15_), di- (C_20_), tri- (C_30_), and tetra-terpenes (C_40_) (Webb *et al.* 2015). The biosynthesis pathways for terpenes are well known and have been studied in several model species [reviewed in Vranová *et al.* (2013)]. Terpenes are derived from the C_5_ precursors isopentenyl diphosphate (IPP) and its isomer dimethylallyl diphosphate (DMAPP). In plants, the mevalonate (MVA) pathway, in the cytosol, produces IPP from acetyl-CoA, some of which is converted to DMAPP by the isopentenyl diphosphate isomerase (IPPI) enzyme (Lange *et al.* 2000; Külheim *et al.* 2011). The methylerythritol phosphate (MEP) pathway, of the chloroplast, produces IPP and DMAPP from pyruvate and glyceraldehyde-3-phosphate. Downstream, the IPP and DMAPP are condensed into prenyl-diphosphates geranyl pyrophosphate (GPP), geranylgeranyl diphosphate (GGPP), and farnesyl pyrophosphate (FPP) which are the immediate substrates for terpene synthesis. The GPP, formed by the union of 1 unit of DMPP and 1 unit of IPP, is the precursor of monoterpenes (C_10_H_16_), FPP, formed by 1 DMPP and 2 IPP’s, is the precursor of sesquiterpenes (C_15_H_24_) and finally, GGPP, formed by 1 DMPP and 3 IPP’s, is the precursor of diterpenes (C_20_H_32_). The 3 molecules GPP, FPP, and GGPP are the substrates used by 1 type of enzymes known as terpene synthases, which catalyze the formation of all terpenes known (Achotegui Castells 2015). Further downstream modification of terpenes often takes place through enzymes such as cytochrome P450 (Pateraki *et al.* 2015), glycosyltransferases (Rivas *et al.* 2013), and methyltransferase (Yauk *et al.* 2015) to produce an even more diverse range of terpenes.

*Eucalyptus* terpenes are stored mainly in schizogenous secretory cavities in the leaf and flower buds (Irmisch *et al.* 2014). The genus *Eucalyptus* contains large amounts of foliar terpenes dominated by monoterpenes, such as 1,8-cineole (Padovan *et al.* 2014), *α*-pinene (Cheng *et al.* 2009), and γ-terpinene (Delaquis *et al.* 2002). Given the variation in terpenes that can occur within a single *Eucalyptus* species, Külheim *et al.* (2015) discovered that *E. grandis* has the largest number of putative functional terpene synthase genes (TPS) compared to any other plant sequenced to date. The TPS family has been split into 7 groups/subfamilies according to phylogenetic analysis namely TPS-a, -b, -c, -d, - e/f, -g, and -h (Bohlmann *et al.* 1997; Chen *et al.* 2011). The TPS can be differentially regulated to generate unique terpene profiles in different parts of the plant or at different times as required. The transcript levels of several terpene synthases increases when plants are wounded (Byun-McKay *et al.* 2006), or suffer herbivory (Beyaert *et al.* 2012) or pathogenic infection (De Alwis *et al.* 2009).

Terpenes influence a wide range of plant–insect interactions often acting as direct and indirect defence against predators. Directly they defer phytophagous insects (De Moraes *et al.* 2001) and indirectly they attract natural enemies (parasitoids and predators) antagonistic to the herbivores (Fiala *et al.* 1994; Takabayashi and Dicke 1996). Terpenes may also act as signals to warn neighboring plants against future insect attacks (Troncoso *et al.* 2012). Gall-inducing insects are some of the most devastating pests and *Leptocybe invasa* Fisher & La Salle (Hymenoptera: Eulophidae) has been causing severe losses on susceptible *Eucalyptus* species or populations within species, resulting in complete failure of some commercially grown clones (Dittrich-Schröder *et al.* 2012).

The use of transcriptomics to investigate gene expression changes following *L. invasa* oviposition has contributed to understanding of the interaction between *Eucalyptus* and *L. invasa* (Oates *et al.* 2015). Significant differences in gene expression induced in response to *L. invasa* oviposition were observed between resistant *E. grandis* (*Egr*) and susceptible *E. camaldulensis* x *E. grandis* (*GC*) clones (Oates *et al.* 2015). The leaves of susceptible clones also had significantly lower amounts of 1,8-cineole and significantly higher amounts of γ-terpinene and *p*-cymene 7 days post oviposition compared to the resistant clones (Oates *et al.* 2015). Naidoo *et al.* (2018) associated terpene profiles with *L. invasa* damage in a *E. grandis* population. Trees highly susceptible to *L. invasa* were associated with increased concentrations of foliar γ-terpinene and *α*-pinene which have been suggested to act as pest attractants, whereas resistant trees were associated with iso-pinocarveol which has been suggested to either have direct effects or act to recruit parasitoids of the pest (Naidoo *et al.* 2018). A subsequent study showed γ-terpinene to act as an attractant to *L. invasa* (Ma *et al.* 2019). These studies have helped in understanding terpene traits that can either act as direct and indirect defences against *L. invasa*.

Several plant phytohormones for example auxins, giberellic acid (GA), salicylic acid (SA), abscisic acid (ABA), and jasmonic acid (JA) regulate terpene biosynthesis pathways (Lv *et al.* 2021). There are several pieces of evidence indicating crosstalk between phytohormones and terpenes (Jogawat *et al.* 2021). JAs are the key phytohormones that mediated crosstalk activities with terpenes. Plant hormones can trigger transcriptional reprogramming leading to a redirection of the metabolic flux toward plant defences including terpenes. Induced responses due to *L. invasa* oviposition included responses such as phytohormones in the 2 genotypes (Oates *et al.* 2015) observed the upregulation of the JA pathway and related genes in response to *L. invasa* oviposition in *Eucalyptus* species. Genes related to JA, SA, ABA pathways were enriched as early as 1 day post oviposition by *L. invasa* and throughout the later time points suggesting that these hormones play important roles as regulators of the plant defence response against *L. invasa* (Oates *et al.* 2021). ABA has been suggested to regulate plant defence against galling insects, for example, there was an increase in ABA levels in galled *Eucalyptus* tissue upon *L. invasa* infestation (Li *et al.* 2017). Increase in SA levels were observed in galled leaves of *E. obliqua* after feeding by gall-forming insect psyllid (Khattab and Khattab 2005). Some studies suggested that hormone concentration in plants may potentially influence gall formation, for example, Li *et al.* (2017) found that galled *Eucalyptus* tissues of *L. invasa* had higher levels of ABA than ungalled tissues during larval feeding suggesting ABA to be a stress response against *L. invasa*.

A breeding program is only viable if there is existing genetically based variation in resistance to a known pest or pathogen. The goal of a Genome-wide association study (GWAS) study is to identify and understand the genetic architecture underlying the phenotypic variation (Tani *et al.* 2020). Several studies (Mendel *et al.* 2004; Durand *et al.* 2011; Dittrich-Schröder *et al.* 2012; Kainer *et al.* 2017; Padovan *et al.* 2017) have shown that significant variation of resistance to the gall wasp and terpenes exists within and between *Eucalyptus* species. Toward this, a recent GWAS in a *E. grandis* breeding population identified single nucleotide polymorphism (SNP) markers and genomic regions on chromosomes 3, 7, and 8 that contained putative candidate genes for resistance against *L. invasa* (Mhoswa *et al.* 2020). The SNP markers explained ∼17.6% of the total phenotypic variation of *L. invasa* resistance. Kainer *et al.* (2019) identified SNP markers for terpene traits including 1,8-cineole, α-pinene, and β-pinene in 468 *Eucalyptus polybractea* genotypes. The study provided novel insights on the genetic architecture of terpene traits and putative candidate genes for breeding of industrially valuable terpenes.

Quantitative (Kainer *et al.* 2017) and qualitative (Padovan *et al.* 2014, 2017) variation in terpene traits have been reported in different *Eucalyptus* species. Qualitative variation in terpenes is likely due to the variation in terpene synthase genes and may be deduced by assessing correlations of terpenes that have the same biosynthetic origin (Keszei *et al.* 2010). A study by Keszei *et al.* (2010) performed a Pearson’s pairwise correlation coefficients between individual terpene concentrations (mg g^−1 ^dry weight DW) from individuals of *Melaleuca alternifolia*. Results showed strong positive correlations between some traits, for example, γ-terpinene and *p*-cymene had a correlation of 0.71. Naghdibadi *et al.* (2017) provided evidence that thymol is biosynthesized by the aromatization of γ-terpinene to *p*-cymene followed by hydroxylation of *p*-cymene. Keszei *et al.* (2008) eluded that γ-terpinene is 1 of 4 possible precursors of *p*-cymene. A hierarchical clustering dendrogram of terpene traits in *E. grandis* showed that γ-terpinene and *p*-cymene clustered close together (Naidoo *et al.* 2018). The likely biological explanation for these results is they share intermediate carbocation (biosynthetically related through the same intermediate precursor) or they are biosynthetically related by descent (Keszei *et al.* 2010).

The use of DNA markers linked to terpene profiles and/or resistance is one of the strategies that can be used in molecular breeding for *L. invasa* resistance. In this study, we aimed to examine the genetic architecture of selected terpene traits and identify putatively associated SNP markers and candidate genes in *E. grandis*.

## Materials and methods

### Genetic materials

**Fig. 1.**
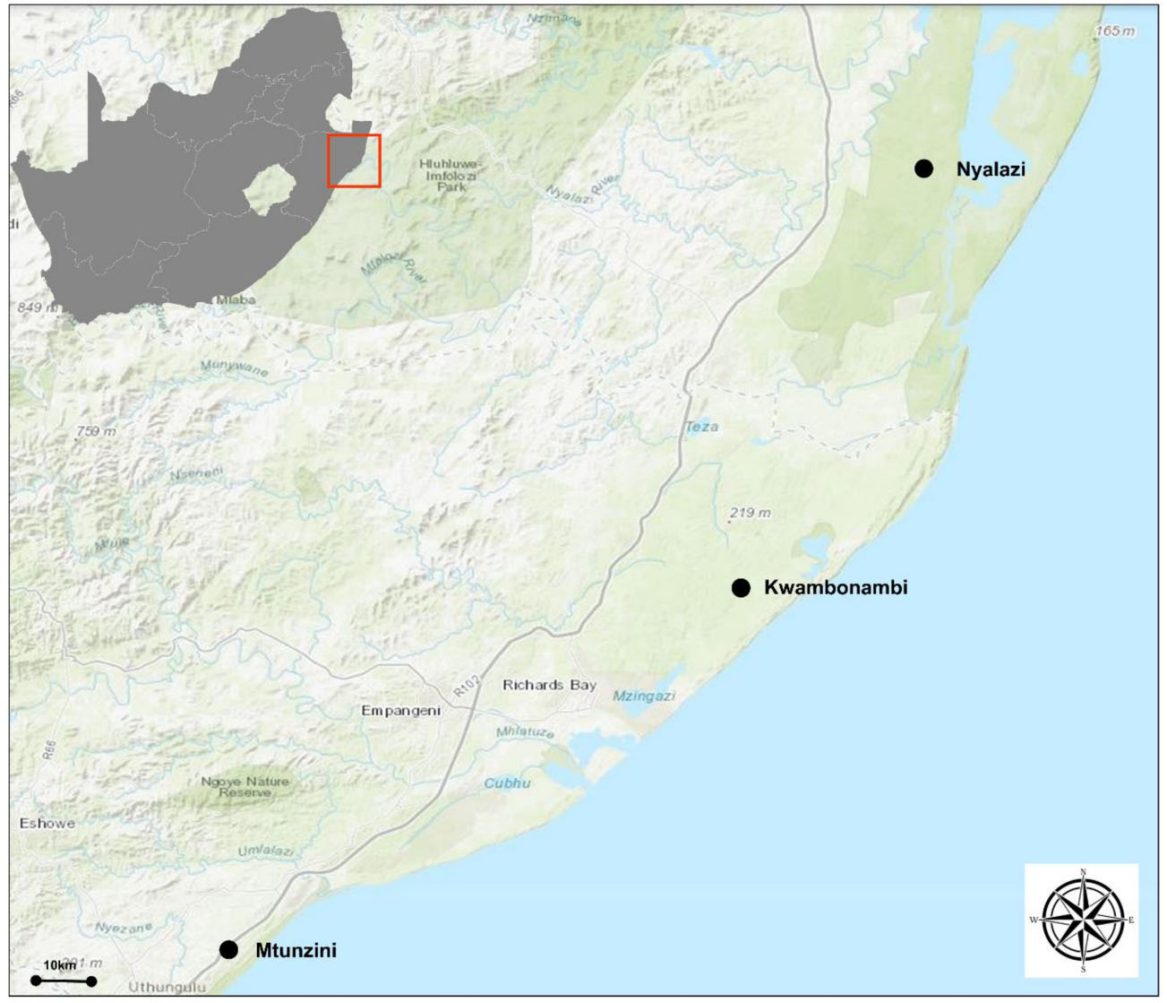
Geographical representation of the trial sites in KwaZulu Natal province, South Africa. The distance (straight line) between Kwambonambi (Siya Quebeka) and Nyalazi is 50 and 66 km between Kwambonambi (Siya Quebeka) and Mtunzini, whereas between Mtunzini and Nyalazi is 112 km.

The genetic material used in this study consisted of 416 *E. grandis* trees from 60 half-sib families randomly sampled from 3 different sites, Kwambonambi (Siya Quebeka), Mtunzini, and Nyalazi in KwaZulu Natal, South Africa as shown in Fig. 1 (Mphahlele *et al.* 2021). The *E. grandis* trials were established in August 2012. In October 2013, when the trees were 14-months old, trees were inspected for *L. invasa* infestation. Symptoms were scored visually using the following scale: 0—not infested, 1—infested showing evidence of oviposition but no gall development, 2—infested with galls on leaves, mid-ribs, or petioles, and 3—stunting and lethal gall formation. Each tree was thus categorized as either 0, 1, 2, or 3. Environmental and trial design information of the study population, the number of families, phenotyped and genotyped individuals are shown in Table 1, the study population used is a subset of a population used by Mphahlele *et al.* (2021). Each family contains 15 individuals (progeny) per trial site, with one of these individuals randomly represented in each of the 15 replications. We have 3 trials sites, therefore each family is represented by 45 individuals across the 3 trial sites. Three to 5 leaf discs measuring 1 cm each were collected from an equivalent position from a side branch on the north side of the tree and placed in pre-weighed vials containing 5 ml of (99.7%) ethanol with tetradecane as internal standard (0.25 mg.l^−1^). The leaf discs were shipped for terpene identification and profiling to the Australian National University (ANU).

**Table 1.** Environmental and trial design information of the study population. The number of families are indicated with the phenotyped and genotyped individuals.

Site		Nyalazi	Kwambonambi (Siya-Quebeka)	Mtunzini
Site environment	Latitude (South)	28^°^ 12’32.01” S	28^°^ 38’ 56.43” S	29^°^ 1’ 52.11” S
	Longitude (East)	32^°^ 20’ 42.79” E	32^°^ 9’ 13.81” E	31^°^ 39’ 23.73” E
	Altitude (m)	47	63	69
	^a^MAP (mm)	999	1196	1220
	^b^MAT (C)	21	21	21
	MAT min. (C)	12	11	11
	MAT max. (C)	30	29	28
Distance (km)	Nyalazi		50	112
	Kwambonambi			66
Trial design	Progeny type	Half-sib	Half-sib	Half-sib
	Trial design	RCB	RCB	RCB
	Replications	15	15	15
	Plot design	Single-tree plot	Single-tree plot	Single-tree plot
Pedigree	*Unrelated families*	33	33	31
	*2nd Gen families*	2	2	
	*3rd Gen families*	32	32	30
	*4th Gen families*	28	28	28
	Total families	95 (61*)	95 (58^*^)	89 (53^*^)
	Number of trees	1890	1830	1680
Survival (%)	Diameter (4yrs)	68	59	58
	*BotryoTera* (3yrs)	83	66	68
	*Lepto*(1.5yrs)	89	80	78
Phenotyped individuals (*n*)	Diameter	1290	1074	970
	*BotryoTera*	1573	1216	1144
	*Lepto*	1687 (123*)	1465 (152*)	1311 (141^*^)
Genotyped individuals (*n*)	Diameter	246	291	278
	*BotryoTera*	321	325	318
	*Lepto*	340 (123*)	358 (152*)	353 (141^*^)

* The number of families, phenotyped and genotyped individuals used in the study.

### Terpene analysis

Three terpene traits 1,8-cineole, γ-terpinene, and *p*-cymene used in this study were obtained from Naidoo *et al.* (2018). The terpenes were measured from 416 *E. grandis* trees using gas chromatography on an Agilent 6890 GC and detected with an Agilent 5973 Mass Spectrometer at ANU as described by Oates *et al.* (2015). Briefly, the Alltech AT-35 column (35% phenyl, 65% dimethylpolyoxylane) (Alltech, Wilmington, DE, USA) was 60 m long with an internal diameter of 0.25 mm and a stationary phase film thickness of 0.25 μm. The carrier gas was Helium, and we injected 1 μl of the ethanol extract at 250 °C at a 1:25 split ratio. The temperature program for the 25 min elution was 100 °C for 5 min, ramping to 200 °C at 20 °C·min^−1^, followed by a ramp to 250 °C at 5 °C·min^−1^, where it remained for 4 min. The peaks were identified by comparing mass spectra to reference spectra in the National Institute of Standards and Technology library (Agilent Technologies, Deerfield, IL, USA) and verified major peaks with authentic standards. One sample from each individual was reanalyzed on the final day of chromatography to confirm that retention times remained stable. The area under each peak was measured manually with the help of MSD Chemstation Data Analysis (Agilent Technologies, Deerfield, IL, USA) and converted to a relative concentration by comparison with the internal standard, tetradecane. The Pearson’s correlation coefficients (*P* ≤ 0.05) was calculated in RStudio v4.0.3 to assess the correlation between each pair of traits based on their concentrations (mg g^−1^ DW) using a method similar to Keszei *et al.* (2010).

### DNA extraction, genotyping, and marker selection

DNA was isolated from 416 *E. grandis* leaf samples using the Machery-Nagel NucleoSpin 96 Plant II kit (Machery-Nagel GmbH & Co. KG, Düren, Germany). The integrity of all DNA preparations was validated by 1% agarose TBE gel electrophoresis and spectrophotometry using a NanoDrop Spectrophotometer (ThermoFisher Scientific, Waltham, MA USA). All samples were genotyped using the EUChip60K SNP chip by GeneSeek (Neogene, Lincoln, NE, USA). Intensity data were first assessed using GenomeStudio V2011.1 (Illumina Inc., San Diego, CA, USA) to recluster genotypic classes as described by Silva-Junior *et al.* (2015). Briefly, SNPs that passed the following criteria were retained, ≥80% samples with GenCall > 0.15, genotype clusters separation > 0.3, mean normalized intensity (*R*) value of the heterozygote cluster > 0.2, and mean normalized theta of the heterozygote cluster between 0.2 and 0.8. SNPs that did not pass these cutoff criteria were removed from further analyses. SNP genotypes were assessed to retain markers ascertained in at least 80% of samples with minor allele frequency (MAF) more than 0.01. Two alternative SNP datasets were also generated by retaining only SNPs with MAF ≥ 0.01 and MAF ≥ 0.05, respectively.

### Linkage disequilibrium, LD pruning, and heritability

Linkage disequilibrium (LD) was evaluated by computing the squared allele frequency correlation coefficient (*r*^2^) using Expectation-Maximization algorithm in SNP & Variation Suite (SVS) v8.4.1 (Golden Helix, Inc., Bozeman, MT, USA; http://www.goldenhelix.com). Average LD was computed for the 11 chromosomes and determined as the *x*-axis intercept of the fitted nonlinear regression of pairwise LD at *r*^2^ = 0.2 against physical distance (in kb) in SVS v8.4.1. LD pruning using a threshold of *r*^2^ = 0.5 was applied on the significant SNP markers by computing the correlations of allele frequencies of significant SNP markers in SVS v.8.4.1. Phenotypic variance was estimated as $\sigma_{p}^{2}=\sigma_{f}^{2}+\sigma_{fS}^{2}+\sigma_{e}^{2}$. Narrow sense heritability was estimated as $h^{2}=3\sigma_{f}^{2}/\sigma_{p}^{2},\;$where $\sigma_{f}^{2}$ is the random effect across families, $\sigma_{fS}^{2}$ is the random effect of family by site interaction, and $\sigma_{e}^{2}$ is the error term. The coefficient of relationship was assumed to be 0.33 instead of 0.25 for half-sib analysis because there is the possibility that some of open-pollinated families were not truly half-sibs, but contained some full-sibs (Squillace 1974). Thus, a coefficient of 3 instead of 4 was multiplied by the family variance in the calculation of heritability.

### Genome-wide association study

GWAS was performed using a stepwise Efficient Mixed-Model Association eXpedited (EMMAX) regression (Kang *et al.* 2008). The method includes significant effects in the model via a forward–backward stepwise approach, while re-estimating the variance components of the model at each step. If the fixed effects included are real, they can reduce the unexplained heritable variance and effectively lower the restraints posed by the mixed model on other markers that correlate with population structure (Segura *et al.* 2012). The significant SNP markers identified in the first step were then assigned as fixed covariates for the second step of GWAS. The stepwise procedure was stopped when no more significant SNP markers were identified. The underlying regression equation for the multilocus mixed model (MLMM) association was: Y=Xβ+Zµ+ɛ, where *Y* is the vector of phenotypes, *X* is the design matrix for the fixed effects (site), *β* is the vector of the fixed effect coefficients (intercept site), *Z* = *Z* is an incidence matrix for the random effects of individual trees, *µ* is the vector of random effect coefficients (genotype, genotype by site interaction), *ε* is the vector of residual effect coefficients. The variance of the random effects was assumed to be $V_{\left(\mu \right)}=\;K{\sigma g}^{2}$ where ${\sigma g}^{2}\;$is the extent to which genetically similar individuals have similar phenotypes or additive genetic variance, K is the variance according to kinship matrix relationship between the random variables, pedigree on which the additive genetic variance is applied to. Manhattan plots for associated SNPs were visualized in Genome Browse v1.0 (Golden Helix, Inc). To select significant SNP markers, different multiple test corrections were applied to the *P*-values obtained using the MLMM approach. A genome-wide adjusted false discovery rate (FDR) set at *P* ≤ 0.05 to correct for multiple testing using the Benjamin–Hochberg procedure and a more stringent genome-wide level of Bonferroni procedure was implemented to control for type I error at *P* ≤ 0.05.

### Candidate genes near associated SNP markers

Using the *E. grandis* reference genome assembly v2 on https://phytozome.jgi.doe.gov, we identified putative candidate genes within a region of 50 kb both upstream and downstream of the SNP markers. Furthermore, we used 2 factors with replication ANOVA and Tukey’s post hoc test with genotype (resistant and susceptible) and treatment (infested and uninfested) as independent variables to determine the effect of gene expression in response to *L. invasa* oviposition. Data (available at plantgenie.org) was obtained from a previous transcriptomics study comparing host responses in a resistant and susceptible background (Oates *et al.* 2015). Briefly, ramets of *Egr* and *GC* were divided into 2 groups, the control group and the test group were exposed to natural infestation by *L. invasa* for 7 days. Infested and uninfested leaves were collected from the control and test groups and total RNA was extracted from the midribs. Total RNA was submitted to the Beijing Genomics Institute (BGI) for RNA-Seq analysis. The reads were mapped to the *E. grandis* genome, assembled into transcripts, and the fragments per kilobase of transcripts per million mapped reads (FPKM) were calculated. Significant differential expression between uninfested and infested samples of each genotype as well as between the uninfested samples and infested samples (i.e. *Egr*-uninfested vs*. GC*-uninfested and *Egr*-infested vs*. GC*-infested) was calculated (Oates *et al.* 2015). Mean differences between and among the variables were considered significant when *P* ≤ 0.05. Statistical analyses were performed using RStudio v4.0.3.

## Results

### Quantitative analysis, heritability, and correlations of terpene traits

**Fig. 2.**
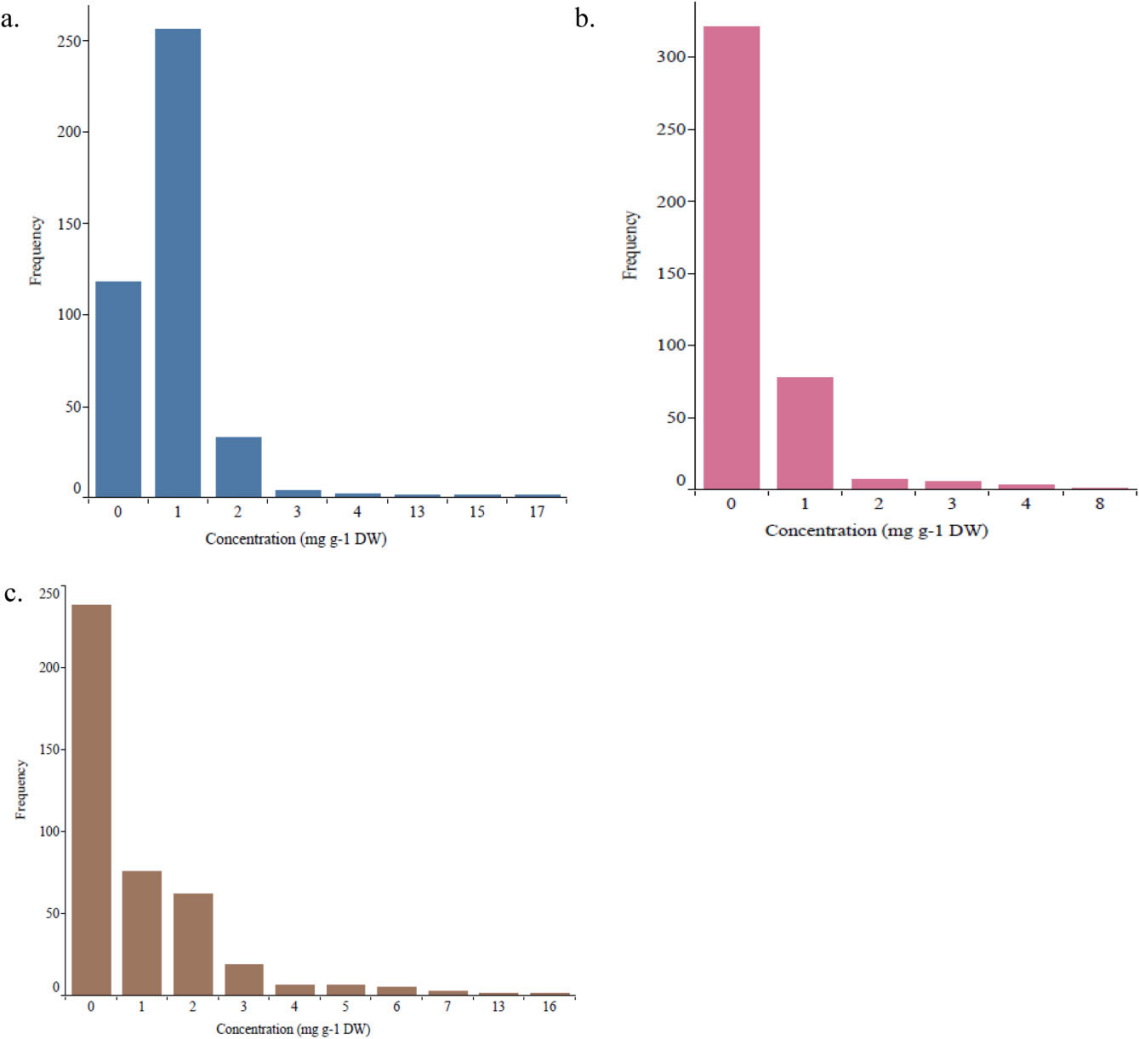
Histograms of foliar terpene concentrations in 416 *E. grandis* trees. Concentration in mg g^−1^ DW of a) 1,8-cineole, b) *γ*-terpinene, and c) *p*-cymene.

Within the 416 *E. grandis* trees, foliar 1,8-cineole concentration ranged from 0 to 17.1 mg g^−1^ DW (average 0.9 mg g^−1^ DW) (Fig. 2a), γ-terpinene ranged from 0 to 8.1 mg g^−1^ DW (average 2.3 mg g^−1^ DW) (Fig. 2b), and *p*-cymene ranged from 0 to 16.8 mg g^−1^ DW (average 1.0 mg g^−1^ DW) (Fig. 2c). The concentration for 1,8-cineole, γ-terpinene, and *p*-cymene was skewed to the right showing that most trees recorded zero or very low concentrations. The terpene distribution of the 3 traits 1,8-cineole, γ-terpinene, and *p*-cymene per site is shown in Supplementary Figs. S1–S3, respectively. The results showed that most trees recorded zero or very low concentrations for the trait γ-terpinene across 3 sites. The trait 1,8-cineole showed a near normal distribution across 3 sites. Heritability for the 3 terpene traits, 1,8-cineole, γ-terpinene, and *p*-cymene showing the genetic parameters (additive and residual variances) for the terpenes at family level across the locations is shown in Supplementary Table 1. There was a moderate positive correlation (*r* = 0.57, *P* < 0.05) between γ-terpinene and *p*-cymene. Weak positive correlations were observed between *p*-cymene and 1,8-cineole (*r* = 0.14, *P* < 0.05) and 1,8-cineole and γ-terpinene (*r* = 0.16, *P* < 0.05) (Supplementary Table 2).

### SNP genotyping, linkage disequilibrium, and population structure

**Fig. 3.**
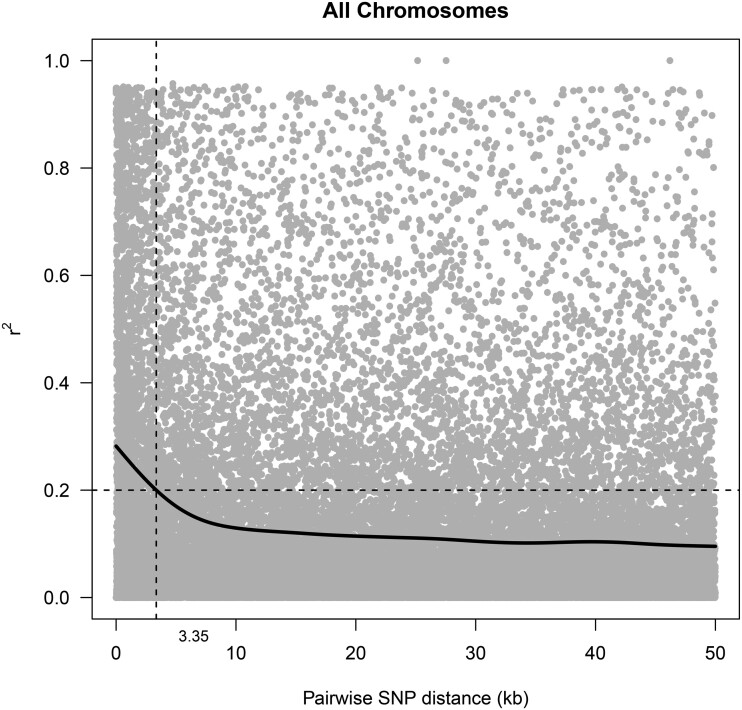
LD decay across 11 chromosomes for 416 *E. grandis* trees within 50 kb distances. The *x*-axis indicates the physical distance between SNP markers within the same chromosome and the *y*-axis indicates the parameter *r*^2^ of LD. The nonlinear regression trend line is shown in black.

Two alternative SNP datasets with different MAF thresholds were used to investigate whether removing lower frequency SNPs had an impact on GWAS results. The genotyping of 416 *E. grandis* trees with 64,639 SNP markers produced a total of 15,387 and 13,770 informative markers in at least 80.0% of the population for MAF ≥ 0.01 and MAF ≥ 0.05, respectively. The resulting SNP density was 1 SNP marker per 39 kb. With LD decaying to *r*^2^ = 0.2 within 3.35 kb on average (Fig. 3), the 15,387 markers tagged 16.2% (103 Mbp) of the 640 Mb *E. grandis* genome (Supplementary Table 3).

**Fig. 4.**
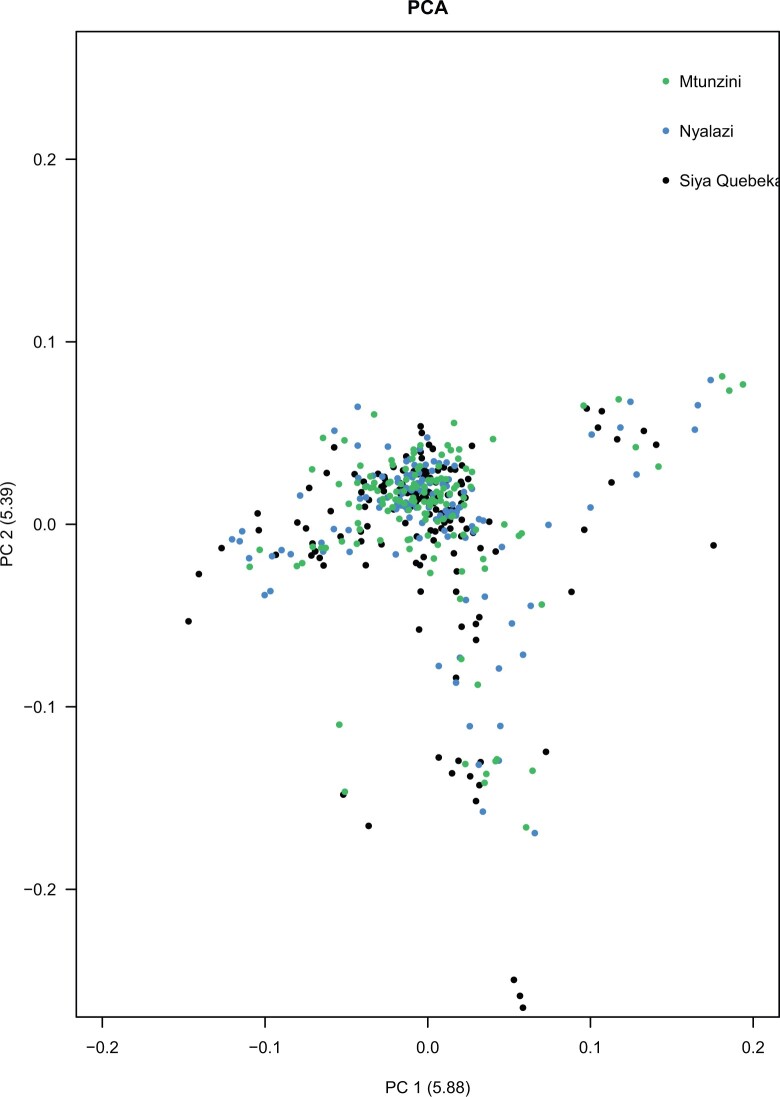
PCA score plot for 416 *E. grandis* population across 3 sites Mtunzini, Nyalazi, and Kwambonami (Siya-Quebeka). The first 2 principal components explained 5.88% and 5.39% of the genetic variance, respectively. The 3 sites Mtunzini, Nyalazi, and Kwambonami (Siya-Quebeka) are shown in green, blue, and black, respectively.

PCA was used to elucidate possible population stratification in the 416 *E. grandis* half-sib population. The first 2 principal components explained 5.88% and 5.39% of the genetic variance, respectively (Fig. 4). The first 2 components explained a small and similar amount of genetic variation suggesting that the population as a whole does not contain strong genetic structure.

### Genome-wide association analysis for 1,8-cineole, γ*-*terpinene, and *p*-cymene

The Quantile-Quantile plots for the 3 terpene traits 1,8-cineole (Supplementary Fig. 4), γ-terpinene (Supplementary Fig. 5), and *p*-cymene (Supplementary Fig. 6) were used to assess the number and magnitude of observed associations between genotyped SNPs and trait under study, compared to the association statistics expected under the null hypothesis of no association. Observed association statistics, −log_10_*P* adjusted values calculated from them, are ranked in order from smallest to largest on the *y*-axis and plotted against the distribution that would be expected under the null hypothesis of no association on the *x*-axis. Since the underlying assumption in GWAS is that the vast majority of assayed SNPs are not associated with the trait, strong deviations from the null suggest a very highly associated locus.

**Fig. 5.**
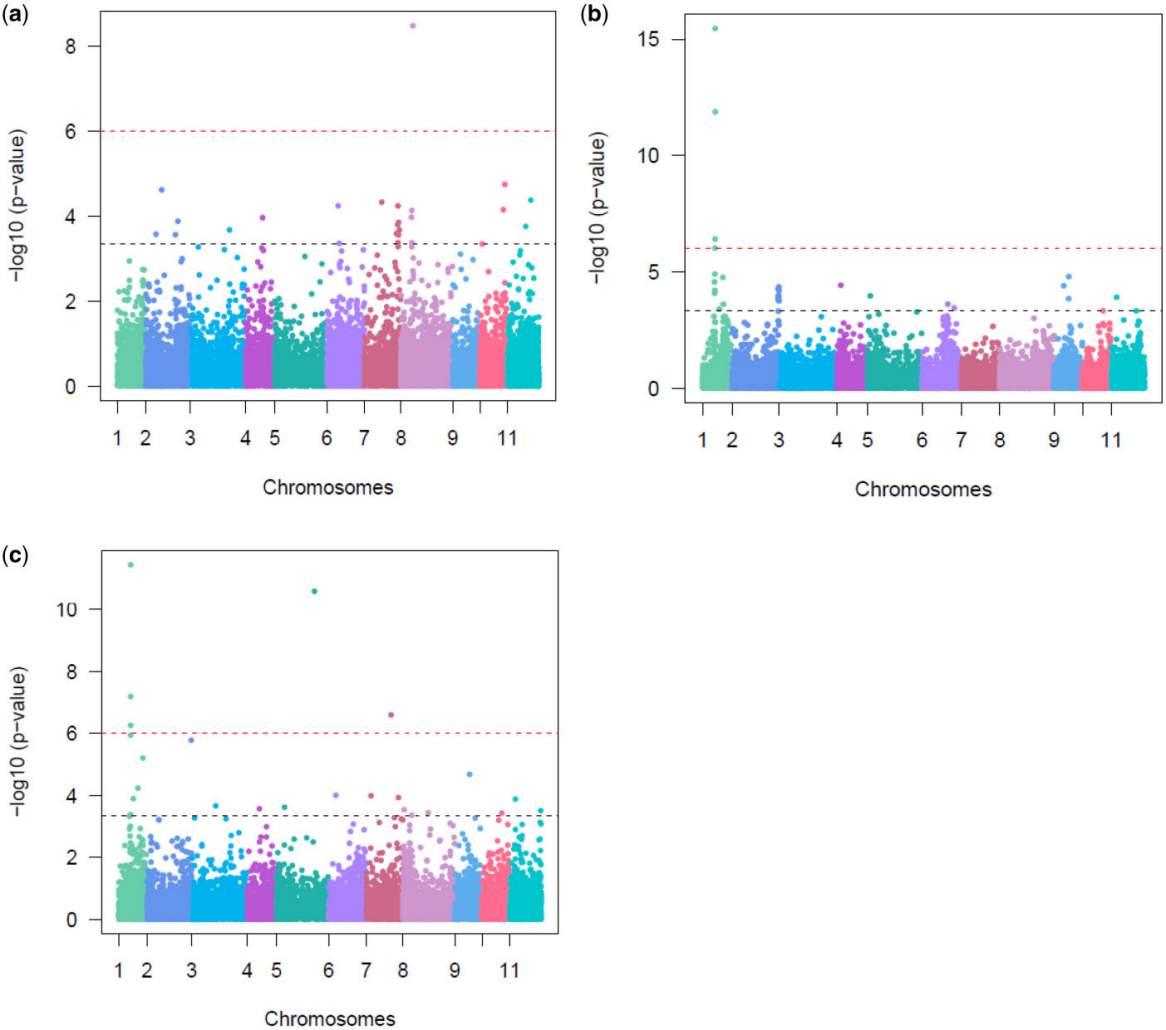
Manhattan plots of −log10 (*P*-value) versus chromosomal position of SNP markers associated with the terpene traits a) 1,8-cineole, b) γ-terpinene, and c) *p*-cymene. The *x*- axis represents chromosomal locations and the *y*- axis, −log_10_ (*P*-values) from genotypic associations. The black dashed horizontal line represents the genome-wide adjusted FDR correction log_10_ (*P*-value = 3.4) and the red dashed line represents the threshold from the Bonferroni correction method (*P*-value = 6).

With a genome-wide adjusted FDR correction –log_10_ (*P*-value) = 3.4 threshold (black dashed line) after multiple testing, the 2 alternative marker datasets MAF ≥ 0.01 and MAF ≥ 0.05 for the 3 traits 1,8-cineole, γ-terpinene, and *p*-cymene did not show any difference in results of the significant SNP marker associated with the trait, therefore we report on the data set MAF ≥ 0.01. The association analysis of 1,8-cineole found 21 SNP markers on chromosomes 2, 4, 6, 7, 8, 9, 10, and 11, and explained between 3.0% and 8.4% of the phenotypic variation (Fig. 5a). Jointly they explained 42.7% of the total phenotypic variation (Supplementary Table 4). When a more stringent adjustment for multiple testing was used, Bonferroni at *P* ≤ 0.05 (red dashed line) only 1 SNP marker was associated with the trait. Association analysis of γ-terpinene found 32 significant SNP markers (Fig. 5b). These SNP markers were found on chromosome 1, 2, 4, 5, 6, 9, and 11, explaining between 3.4% and 15.5% of variation each, and jointly they explained 54.5% of the total phenotypic variation (Supplementary Table 5). For γ-terpinene with Bonferroni at *P* ≤ 0.05, 4 significant SNP markers were associated with the trait. The association analysis of *p*-cymene found 28 significant SNP markers (Fig. 5c). The SNP markers were found on chromosomes 1, 2, 3, 5, 6, 7, 10, and 11, explaining between 3.4% and 16.1% of the variation each, and jointly they explained 46.9% of the total phenotypic variation (Supplementary Table 6). For *p-*cymene with Bonferroni at *P* ≤ 0.05, 5 significant SNP markers were associated with the trait. Our results showed that γ-terpinene and *p*-cymene had 8 SNP markers in common (Supplementary Tables 5 and 6).

### Candidate genes for foliar 1,8-cineole, γ-terpinene, and *p*-cymene concentrations

Based on the detailed annotation for the *E. grandis* reference genome, putative candidate genes of the significant SNP markers were identified within the 100 kb (50 kb upstream and 50 kb downstream) of the significant SNP associations with the 3 terpene traits 1,8-cineole, γ-terpinene, and *p*-cymene. The number of genes identified were 166, 270, and 216 (Supplementary Tables 4–6), respectively. The study reported on genes we suggest could play a role in terpene biosynthesis. Eucgr.F00752, a candidate gene for *p*-cymene encodes geranylgeranyl diphosphate synthase (*ggpps*) tagged by the SNP marker EuBR06s10733103 and explains 3.66% of the phenotypic variation. Eucgr.H01393, a candidate gene for 1,8-cineole encodes isopentenyl pyrophosphate isomerase 2 (*ippi*) tagged by the SNP marker EuBR08s17426789 explains 8.48% of the phenotypic variation. A cluster of UDP-glycosyltransferase genes (Eucgr.B03996, Eucgr.B03997, Eucgr.H01240, Eucgr.H01241, Eucgr.H01242, Eucgr.H01243, Eucgr.H01244, Eucgr.H01245, and Eucgr.H01247) were identified for γ-terpinene and 1,8-cineole traits, the SNP markers EuBR02s63883704 and EuBR08s15122213 tagging these gene were found on chromosome 2 and 8 explaining 3.76% and 3.97% of the phenotypic variation, respectively, were identified. Our study identified ubiquitin protein ligase genes Eucgr.A01473, Eucgr.A01934, Eucgr.A01937, Eucgr.B03986, Eucgr.G03167, leucine-rich repeat protein kinase Eucgr.B02179, Eucgr.G02950, Eucgr.J02704, Eucgr.J02705 as well as an expansin gene Eucgr.A01806. The SNP makers tagging these genes explained between 3.47% and 4.88% of the phenotypic variation. Genes related to phytohormone were identified, for example, Eucgr.G01437 and Eucgr.K02605 encoding an abscisic acid-responsive (TB2/DP1, HVA22) family protein. The SNP markers EuBR07s24890821 and EuBR11s33763867 tagging these 2 genes explained 4.33% and 4.88% of the phenotypic variation. Eucgr.G01954 a candidate gene for *p*-cymene encodes jasmonate-ZIM domain protein (JAZ). The SNP marker EuBR07s35438867 tagging this gene was found on chromosome 7 explaining 4.06% of the phenotypic variation. The genes Eucgr.G01952 for *p*-cymene and 2 genes Eucgr.K00658 and Eucgr.K00660 for γ-terpinene each encode auxin family protein. The SNP markers tagging these genes explained 4.06% and 3.91% of the phenotypic variation, respectively.

**Fig. 6.**
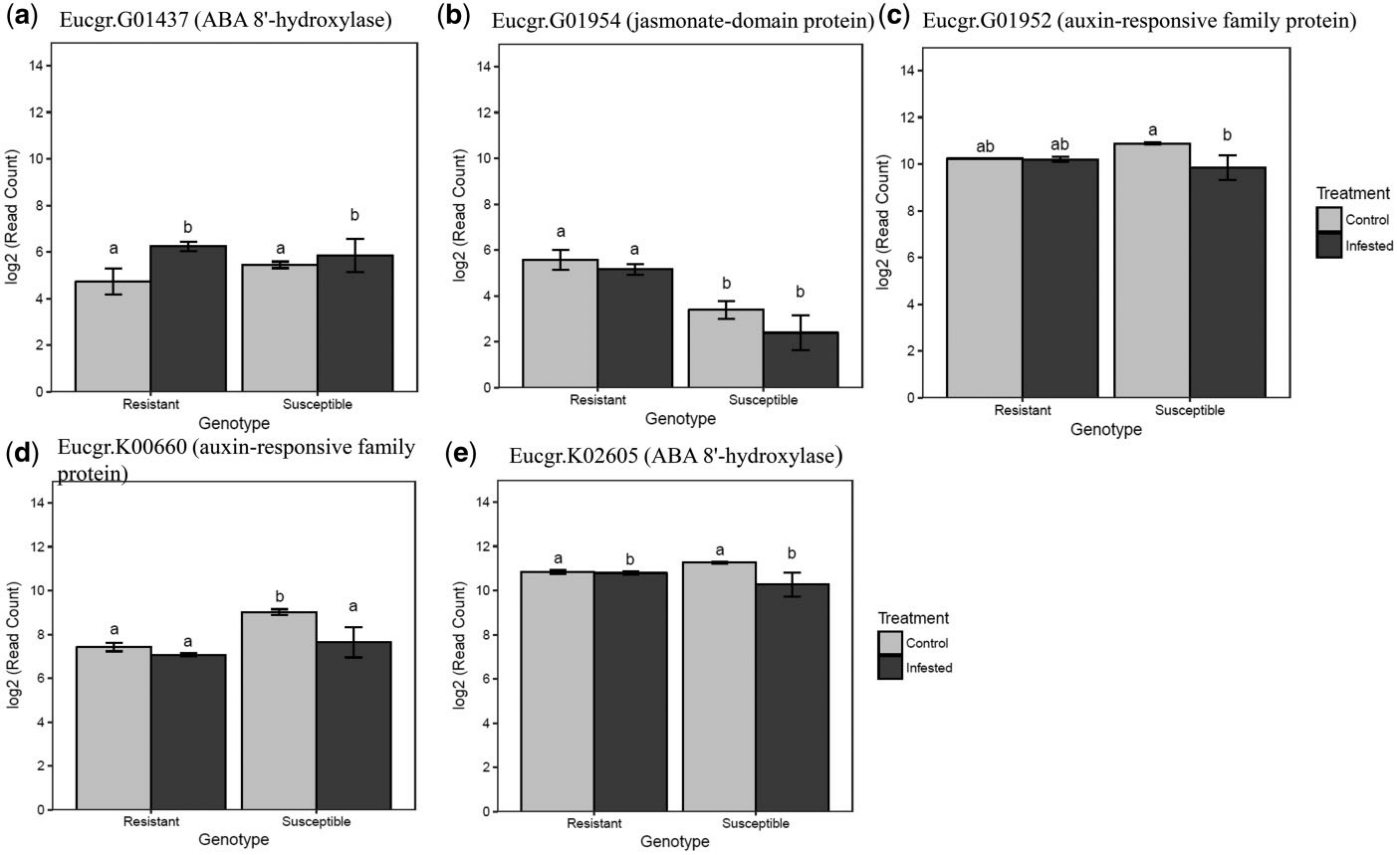
The effects of mean gene expression (± 1 SD) within and between genotypes of each of the 5 candidate genes (a–e) for the genes that play a role in phytohormone biosynthesis identified as significant by ANOVA and post hoc tests. Means sharing letters were not significantly different, light gray are the control and dark gray infested with *L. invasa*.

All putative candidate genes were investigated for differential expression resistant *E. grandis* (*Egr*) and susceptible *E. camaldulensis* × *E. grandis* (GC) from an independent experiment (Oates *et al.* 2015). We show the expression patterns of genes involved in phytohormone biosynthesis. This provides another line of evidence for a possible association between terpene traits and response to infestation. In addition, Oates *et al.* (2015) showed expression patterns of genes involved in phytohormone biosynthesis and the use of this previously generated gene expression data in our study was to provide basic information on the candidate genes, that is, whether the genes are expressed, induced, or repressed. The expression patterns of genes involved in phytohormone biosynthesis is shown in Fig. 6. The expression pattern of 1 gene Eucgr.G01954 (Fig. 6b) was identified as significant within or between genotypes. In some cases, the results indicate that gene expression is altered in the same manner between resistant and susceptible genotypes, for example, Eucgr.01437 (Fig. 6a). Or that the susceptible genotype responds to *L. invasa* oviposition such that the resultant gene expression is not significantly different from the resistant, Eucgr.K00660 and Eucgr.K02605 (Fig. 6d and e). The results of the ANOVA showing the effects of mean gene expression within and between genotypes of each of the candidate genes are shown in Supplementary Table 7.

## Discussion

This study aimed to examine the genetic architecture of terpene traits and identify putative candidate genes important for *L. invasa* resistance in *E. grandis* by using medium density SNPs 15,437 from a multispecies EUChip60K SNP chip and a moderate population size of 416 individuals. We acknowledge that the moderately small population size meant that statistical power to detect loci of smaller effect was limited (Visscher *et al.* 2017). The use of the EUChip60K SNP chip determined the genome coverage as the number of polymorphic SNPs vary according to the species diversity, while the specific population under analysis determines the LD available for marker association. The average LD of 3.35 kb around each of the 15,437 polymorphic SNPs means that the study was able to detect marker associations in up to 103 Mb (16%) of the 640 Mb *E. grandis* genome. We speculate that perhaps LD is so much higher in some of these associated loci hence regardless of the low genome coverage, we were able to detect major loci explaining large proportion of the phenotypic variation. This suggests that our study likely missed many polymorphisms contributing to the phenotypic variation and that there is still potential to detect many marker associations in this study population using other genotyping approaches. For example, Kainer *et al.* (2019) used a whole-genome sequencing approach with approximately 2.3 million SNP markers, providing comprehensive genome coverage and finer resolution for marker-trait association. Nevertheless, results obtained from this study were valuable in detecting marker-trait associations for terpene traits in *E. grandis* and identifying putative candidate genes associated with the trait.

Results showed that the foliar concentrations of the 3 terpene traits were skewed to the right (Fig. 2a–c) indicating that most trees recorded zero or very low concentrations. Strong associations were observed, which may suggest that the few individuals with greater than zero values do carry the allele(s) associated with the trait. A moderate positive Pearson’s correlation of 0.57 was observed between γ-terpinene and *p*-cymene and these 2 traits shared 8 significant makers in common. Kessler *et al.* (2010) reported a strong positive correlation of 0.71 between γ-terpinene and *p*-cymene in *Melaleuca alternifolia*. Keszei *et al.* (2010) provided evidence that *p*-cymene is made from γ-terpinene. Hierarchical clustering dendrogram of terpene traits in *E. grandis* showed that γ-terpinene and *p*-cymene clustered close together (Naidoo *et al.* 2018). These results suggest that γ-terpinene and *p*-cymene might be biosynthetically related through same intermediate precursor or related by descent. The more γ-terpinene there is, the more *p*-cymene can be made from it, though *p*-cymene has additional potential precursors (Keszei *et al.* 2010).

The SNP markers explained variation that was generally within the range of SNP markers for other quantitative traits in *E. grandis* (1–5%) (Resende *et al.* 2017). The existence of SNP markers that explain less than 5% variation suggests that alleles of small effects may play a role in determining phenotypes in terpene traits in *E. grandis* (Supplementary Tables 4–6). SNP markers explaining more than 5% of the phenotypic variation were observed for the 3 traits 1,8-cineole, γ-terpinene, and *p*-cymene with the strongest association of the SNP marker EuBR01s17197369, explaining 15.46% and 16.08% of the phenotypic variation for γ-terpinene and *p*-cymene, respectively (Supplementary Tables 5 and 6). The results suggest that terpene traits are influenced by a few large-effect loci in combination with minor effect loci (i.e. an oligogenic trait, or possibly polygenic if all the associations in this population could be detected).

A previous GWAS by Kainer *et al.* (2019) revealed the association of some terpene traits with biosynthetic pathway genes and identified a terpene synthase gene cluster on chromosome 1. We observed that some SNP markers identified on chromosome 1 in our study were in proximity with the SNP markers tagging terpene synthase genes in Kainer *et al.* (2019), for example, our SNP marker EuBR01s28423985 associated with γ-terpinene and *p*-cymene on position 33,057 Mb is approximately 14 kb away from the SNP marker in chromosome 1 on position 33,044 Mb tagging a terpene synthase gene EgranTPS063. Our study did not find any terpene synthase gene within 100 kb of the SNP marker EuBR01s28423985. Although, the results suggest that these SNP markers could be associated with the same genetic factor, this is impossible to test since different species, that is, *E. grandis* versus *E. polybractea*, different genotype platforms, whole-genome sequencing versus EUChip60K SNP chip were used in the 2 studies, and the window region for candidate gene search was different between the 2 studies.

The identification of the candidate genes in a window region (100 kb) around significant SNP markers was based on the observed LD decay in this study and the known gene density in the *E. grandis* genome. The use of previously generated gene expression data in our study (Oates *et al.* 2015) was to provide basic information on the candidate genes, whether the gene are expressed, induced, or repressed. Gene expression changes merely indicate that the transcript levels are perturbed during the infestation. This provides 1 line of evidence for a possible association, but many lines of evidence will be required to establish whether any of these genes are directly or indirectly involved in the response.

Two candidate genes identified within 100 kb of a significantly associated SNP are known biosynthetic pathway steps in terpene biosynthesis. The first, *geranylgeranyl diphosphate synthase* (*ggpps*), Eucgr.H01235 located near SNP EuBR06s10733103. The enzyme GGPPS uses IPP and DMAPP as substrate and produces GGPP, the substrate for di-terpene synthases and for tetraterpene biosynthesis. One study found a significant association between GGPPS and beta-carotene (a tetraterpene) in *Sorghum bicolor* (Cruet‐Burgos *et al.* 2020). Külheim *et al.* (2011) found a significant association between GGPPS and the ratio of mono- to sesquiterpenes in *E. globulus*. By using IPP and DMAPP as substrates, GGPPS reduces the available precursors for monoterpene synthesis and could hence influence the concentration of foliar monoterpenes (Külheim *et al.* 2011). The second candidate gene is *isopentyl diphosphate isomerase* (*ippi*), Eucgr.H01393, was located near the significant marker EuBR08s17426789. The enzyme IPPI isomerizes IPP to its structural isomer DMAPP, thereby changing the ratio of IPP: DMAPP, which determines which prenyldiphosphate synthase (GPPS, GGPPS in the chloroplast, or FPPS in the cytosol) is most active since they all use different ratios of IPP: DMAPP (Phillips *et al.* 2008; Webb *et al.* 2015). Most plants, including *E. grandis*, have 2 copies of this gene, which may allow different subcellular targeting and regulation of gene expression (Wildung and Croteau 2005; Jin *et al.* 2020). Webb *et al.* (2013) found a slight correlation between the gene expression of *ippi1* and several foliar monoterpene concentrations in *Melaleuca alternifolia*, and some stronger correlation between gene expression of *ippi2* and several mono- and sesquiterpene concentrations as well as the ratio of mono- to sesquiterpenes. A candidate gene association study in *Eucalyptus loxophleba* found 1 SNP in *ippi* associated with both 1,8-cineole and α-pinene at the significance threshold of *P* = 0.05 (Padovan *et al.* 2017).

A cluster of UDP-Glycosyltransferase genes associated with the terpene traits, 1,8-cineole, γ-terpinene, and *p*-cymene were identified. These genes may be involved in terpene transport and storage. The putative *A. thaliana* ortholog AtUGT87A2 for the gene Eucgr.B03996 and Eucgr.B03997 glycosylates monoterpenes which accumulate throughout flowering, leading to considerable storage of potential aroma constituents that account for most nonvolatile aroma compounds (Wang *et al.* 2012). The UGT72E2 (AT5G66690) and UGT72E1 (AT3G50740) putative *A. thaliana* orthologs for the genes Eucgr.H01242, Eucgr.H01243 and Eucgr.H01244 encodes glycosyltransferases shown to glucosylate several terpenes in vitro (Lanot *et al.* 2006, 2008). Kainer *et al.* (2019) reported on AtUGT85A2 a putative *A. thaliana* ortholog for the genes Eucgr.J00971, Eucgr.J00972, and Eucgr.J00973, which showed strong activity with citronellol and geraniol suggesting that it plays a role in the glycosylation of monoterpene which may influence their concentration. Glycosylation of terpenes enables transport and storage of hydrophobic terpenes (Rivas *et al.* 2013; Schwab *et al.* 2015), which in turn may have an effect on foliar monoterpene or total terpene concentration. Terpene glycosylation has been shown to occur naturally in many plants, including cultured *Eucalyptus perriniana* cells (Shimoda *et al.* 2006).

Eucalypts terpenes are stored mainly in schizogenous secretory cavities in the leaf and flower buds (Irmisch *et al.* 2014). A study by Ishizaki (2015) found 2 proteins in a U-box E3 ubiquitin ligase, which interacts with a leucine-rich repeat kinase that play a role in the initial formation of schizogenous secretory cavities in common liverwort, *Marchantia polymorpha*. Our study identified ubiquitin protein ligase genes Eucgr.A01473, Eucgr.A01934, Eucgr.A01937, Eucgr.B03986, Eucgr.G03167, leucine-rich repeat protein kinase Eucgr.B02179, Eucgr.G02950, Eucgr.J02704, Eucgr.J02705 as well as an expansin gene Eucgr.A01806 associated with the terpene traits 1,8-cineole, γ-terpinene, and *p*-cymene. Our results are in accordance with the study by Kainer *et al.* (2019) who identified a U-box E3 ubiquitin-protein ligase Eucgr.F00384, a leucine-rich repeat kinase Eucgr.G02301 and an expansin-like gene Eucgr.E00317. Kainer *et al.* (2019) speculated that these genes may work together to form cavity structures. These are lines of evidence for an involvement of these genes in cavity formation, however, functional studies are required to verify their involvement in cavity formation.

Some studies have provided evidence on the crosstalk between phytohormones and terpenes (Jogawat *et al.* 2021). Our study identified putative phytohormones candidate genes within the 100 kb of a significantly associated SNP markers for the terpene traits 1,8-cineole, γ-terpinene, and *p*-cymene. The Eucgr.G01437 gene (*A. thaliana* ortholog AT3G19270), a member of the CYP707A gene family encoding a protein with ABA 8′-hydroxylase activity and Eucgr.K02605 (putative *A. thaliana* ortholog AT5G42560) encoding abscisic acid (TB2/DP1, HVA22) family protein are involved in ABA synthesis. ABA was suggested to initiate defence in host plants (Tooker and De Moraes 2011; Erb *et al.* 2012). Oates *et al.* (2015) found that genes related to ABA showed differential expression between susceptible and resistant *Eucalyptus* varieties after infestation by *L. invasa*. However, some studies suggested that ABA concentration in plants may potentially influence gall formation (Tooker and De Moraes 2011; Tokuda *et al.* 2013). ABA concentration was found to be higher in the galls made by *Lipara lucens*, *Dryocosmus kuriphilus*, and *Schisandra chinensis* (Wood and Payne 1988; Csoka 1998; Wang *et al.* 2016). Li *et al.* (2017) found that galled *Eucalyptus* tissues of *L. invasa* had high concentrations of ABA than ungalled tissues during larval feeding, suggesting ABA to be a stress response against *L. invasa*. Depending on the interaction between the host and pest, it can be suggested that ABA plays a role in the regulation of defence against *L. invasa* or contributes to the initiation of gall formation in susceptible *Eucalyptus* genotypes.

Eucgr.G01954 encodes a jasmonate-ZIM domain protein (JAZ). JA is considered the major regulator of plant resistance to insect oviposition (Cooper and Rieske 2011; Reymond 2013). Oates *et al.* (2015) observed the upregulation of the JA pathway genes in response to *L. invasa* oviposition in *Eucalyptus* species. The *Brassica rapa* ortholog Bra008846 for *A. thaliana* AT5G13220 (JAZ1, JAZ10, TIFY9) gene was upregulated in the JA pathway upon wounding (Saha *et al.* 2016) and this *A. thaliana* gene AT5G13220 is an ortholog for *E. grandis* gene Eucgr.G01954. Chung *et al.* (2008) investigated the regulation and function of *JAZ* genes during the interaction of *A. thaliana* with the generalist herbivore *Spodoptera exigua*. Results showed that most members of the *JAZ* gene family were highly expressed in response to *S. exigua* feeding and mechanical wounding suggesting the role played by JA in defense.

Auxins regulate several plant processes including growth and development as well as defence against pest and pathogen attack (Mano and Nemoto 2012; Yang *et al.* 2014; Glick 2015). In other studies, auxins have been shown to be involved in insect gall development across interactions involving different species (Bartlett and Connor 2014; Tooker and Helms 2014; Takeda *et al.* 2019). The genes Eucgr.G01952 with putative *A. thaliana* ortholog AT1G12820 (AFB3), Eucgr.K00658 with putative *A. thaliana* ortholog AT2G04850, and Eucgr.K00660 with putative *A. thaliana* ortholog AT5G47530 encode for auxin-responsive family proteins. Oates *et al.* (2015) observed an upregulation of auxin metabolism-related genes in the susceptible clone. These results suggest that auxin may contribute to the production of gall-specific tissues in the susceptible interaction. There were no differences in the expression of the gene in the resistant infested and control genotypes, but a difference was observed in the susceptible genotype where its role is less clear. In the susceptible interaction, manipulation by *L. invasa* may cause the observed changes in gene expression, there is no concrete evidence to support this (Fig. 6c). However, a study by Bedetti *et al.* (2017) showed that accumulation of auxins in the galls of susceptible *Piptadenia gonoacantha* (Fabaceae) played a role in influencing the size, shape, different direction of cell expansion and these changes may influence gene expression.

## Conclusion

Even though our study had limited genome coverage for GWAS (∼16%), the associations that we detected adds to our understanding of the genetic architecture that underlies terpene traits in *E. grandis*. We showed that variation in the 3 terpene traits 1,8-cineole, *γ*-terpinene, and *p*-cymene may be influenced by at least a few loci of minor and a few of major effect, suggesting an at least oligogenic nature of the traits (possibly polygenic if all the undetected loci are included in future). The results from the study provide a list of SNP markers and putative candidate genes, for example, IPPI and GGPPS that were previously reported to be involved downstream of the MVA and MEP pathways in terpene production (Külheim *et al.* 2011). These results warrant further investigation to provide additional information on the genetic architecture of terpene traits for *E. grandis*. Future studies will include testing and validation of the associated loci in different *Eucalyptus* species.

## Data availability

All datasets used in GWAS analysis are available at https://figshare.com/s/92751d332fd45a1b58f1. Data of the results obtained from the GWAS analysis was used to generate the figures Manhattan plots, LD decay, and PCA plot. Gene expression data were obtained from a previous study by Oates *et al.* (2015). The raw data are available through the NCBI (BioProject PRJNA305347), https://www.ncbi.nlm.nih.gov/bioproject/PRJNA305347. Resistant infested (*Egr* TAG 5 infested: SRA, SRX1470128), resistant uninfested (*Egr* TAG 5 uninfested: SRA, SRX1470127), susceptible infested (*Egr* GC540 infested: SRA, SRX1471865), and susceptible uninfested (*Egr* GC540 uninfested: SRA, SRX1470184). The FPKM is available at https://figshare.com/s/92751d332fd45a1b58f1.

Supplemental material is available at *G3* online.
